# Relationship of D-dimer and prediction of pulmonary embolism in hospitalized COVID-19 patients: a multicenter study

**DOI:** 10.2217/fmb-2021-0082

**Published:** 2021-07-28

**Authors:** Iftikhar Nadeem, Asad Anwar, Louise Jordon, Noor Mahdi, Masood Ur Rasool, Jonathan Dakin, She Lok

**Affiliations:** ^1^Department of Respiratory Medicine, East & North Hertfordshire NHS Foundation Trust, Stevenage, UK; ^2^Department of Respiratory Medicine, Royal Surrey Hospital NHS Foundation Trust, Guildford, Surrey, UK; ^3^Department of Respiratory Medicine, Norfolk & Norwich University Hospital NHS Trust, Norwich, UK

**Keywords:** computerized tomographic pulmonary angiography, CTPA, COVID-19, D-dimer, optimal threshold analysis, pulmonary embolism

## Abstract

**Aim:** COVID-19 is a known risk factor for pulmonary embolism (PE). In this retrospective, multicenter study, we aimed to determine an optimal D-dimer cutoff to predict PE in hospitalized patients with COVID-19. **Materials & methods:** A total of 193 patients underwent computerized tomographic pulmonary angiography imaging and were classified into PE positive and negative groups. Physiological, radiological and biochemical parameters were compared and receiver operator curve analysis was conducted to determine a predictive D-dimer threshold. **Results:** An optimal D-dimer cutoff of 2494 ng/ml was selected (Youden index: 0.906), giving a sensitivity of 100% (95% CI: 100–100) and specificity of 90.62% (95% CI: 90.5–90.8) for predicting PE. **Conclusion:** We propose that in the absence of other clinical signs, a D-dimer threshold of 2495 ng/ml could be used with high sensitivity and specificity to predict PE in hospitalized patients with COVID-19.

The SARS-CoV-2 was detected in patients hospitalized with pneumonia in Wuhan, China [1]. WHO declared this novel coronavirus disease, COVID-19, a global pandemic on 11 March 2020 [2]. As of 8 March 2021, there have been 116,8521,281 confirmed COVID-19 cases worldwide including 2,589,548 deaths around the globe [3].

SARS-CoV-2 enters the host cells by binding with angiotensin converting enzyme 2 receptor (ACE 2) through its structural spike protein (S). Viral uptake is promoted by type 2 transmembrane serine protease (TMPRSS2), present in host cells, by cleaving the ACE 2 and hence activating spike (S) protein [4]. The inflammatory response to SARS-CoV-2 consists of both innate and adaptive immune response, debilitated lymphopoiesis and increased lymphocyte degradation. In later stages of illness, when viral replication accelerates, it can cause pulmonary endothelial barrier damage, impaired alveolar-capillary oxygen transmission and impaired gas exchange [5].

Severe COVID-19 is considered a prothrombotic state [6,7]. Klok *et al.* reported a 31% incidence of thrombotic complications in critically ill ICU patients with COVID-19, most commonly pulmonary embolism (PE) [6]. Notably the symptoms of PE and severe COVID-19 are clinically challenging to distinguish. Furthermore, one of the most typical feature of coagulopathy reported in COVID-19 is elevated D-dimer concentration [8–11]. D-dimer levels have been shown to correlate with disease severity and to be a reliable prognostic marker for in-hospital mortality [10].

So far, no single cut-off value of elevated D-dimer concentration has been agreed upon, as threshold for further radiological investigations to exclude venous thromboembolic complications. The International Society of Thrombosis and Hemostasis considered that a three–fourfold increase in D-dimers concentration may be significant, in interim guidance for recognition and management of coagulopathy in COVID-19 [11]. Likewise, the association of high D-dimer concentration with severity of PE is unknown. Moreover, the role of the Wells score in evaluation of venous thromboembolic complicating COVID-19 is also less clear in the setting of COVID.

## Materials & methods

### Study setting & patient selection

This was a retrospective, multicenter study in two district general hospitals in England. We included all patients hospitalized from 1 November 2020 to 31 January 2021 with proven COVID-19 pneumonia and D-Dimer concentration, who underwent computerized tomographic pulmonary angiography (CTPA) due to clinical suspicion of PE. Patients were followed until death or 31 January 2021. Laboratory confirmation of SARS-CoV-2 was defined as a positive result of real-time reverse transcriptase-PCR assay of nasal and pharyngeal swabs. Management of COVID-19 was at the discretion of the physicians in charge. In line with prior studies of this nature, patients on prior anticoagulant therapy were not excluded from the study cohort [12–15].

### Data collection

Baseline characteristics, observations on arrival (respiratory rate, oxygen saturations, heart rate and blood pressure) were recorded for each placement. Data for white cell count (WCC), C-reactive protein (CRP), D-dimer levels, creatinine, fibrinogen, platelets, international normalized ratio (INR), prothrombin time and Well’s score were collected for each patient. All blood tests were taken at admission. Latex agglutination assay was used to measure D-dimer levels at both sites. CTPA findings were recorded (as documented in the report by the site radiologists), including presence of absence of PE and clot burden (quantified by bilateral or unilateral PE findings). Average time interval between admission and CTPA was 36 h. Dalteparin was given both as prophylaxis and treatment of PE.

### Study objectives

The primary objective of our study was to determine a D-dimer threshold for predicting PE in COVID-19 patients which optimizes sensitivity and specificity and, hence, can be used with confidence in clinical practice. The secondary objectives were: to explore the value of Well’s score in predicting PE in COVID-19 patients; to explore associations between PE and other potential predictive measures (respiratory rate, CRP, WCC, creatinine, lymphocyte count, fibrinogen, platelets, INR and prothrombin time); and to determine any relationship between CRP and D-Dimers in COVID-19 patients.

### Statistical measures

All statistical tests were performed using Statistical Package for the Social Sciences version 16. Appropriate parametric and nonparametric measures were utilized based upon the data distribution. Where appropriate, two level p-values (p < 0.05) and 95% CI were calculated to determine significance. Receiver operating characteristic (ROC) curve analysis was performed and the Youden Index calculated to determine the optimal D-dimer threshold to predict PE.

### Ethical approval

We sought approval from local research and development board at both sites. In view of retrospective nature of the study, a formal ethical approval was not required, confirmed with the research and development board of both institutes. Results are reported in accordance with the Strengthening the Reporting of Observational Studies in Epidemiology guidelines. Principles outlined in the Declaration of Helsinki have been followed for this study.

## Results

**Figure 1.**
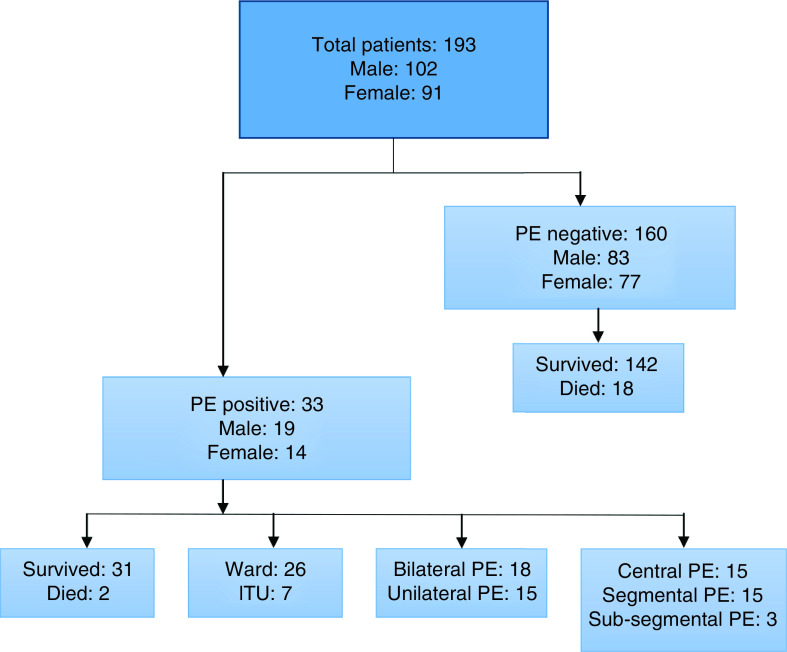
Flow chart showing characteristics of study population. ITU: Intensive treatment unit; PE: Pulmonary embolism.

From 1 November 2020 to 31 January 2021, 193 patients presented to the two study facilities and met the inclusion criteria. A total of 102 patients were male and 91 were female, with a median age of 59 years. Overall, 33 patients (17%) had a PE on CTPA imaging. Nine patients were taking anticoagulation at hospital presentation. In total, 20 patients in the study population died during their inpatient admission; 18 patients in the PE-negative group and two patients in the PE-positive group (Figure 1). The characteristics of both groups are displayed in Table 1.

**Table 1.** A table to show the descriptive, radiological, physiological and biochemical parameters of the study population.

Characteristic (on admission)	PE-positive patients (n = 33)	PE-negative patients (n = 160)
Male	19	83
Female	14	77
Age (median)	67	58
Anticoagulant treatment on admission	1	8
Radiological evidence of pneumonitis	30	134
Respiratory rate (mean, breaths/minute)	25.6 (95% CI 23.9–27.3)	21.9 (95% CI 21.2–22.7)
Oxygen saturation via pulse oximetry (mean, %)	82.6 (95% CI 81.5–83.7)	89.1 (95% CI 87.4–90.8)
Temperature (mean, C)	36.8 (95% CI 36.6–37.0)	37.5 (95%Ci 36.9–38.1)
Heart rate (mean, beats per min)	98.7 (95% CI 92.6–105)	95.9 (95% CI 93.6–98.2)
Systolic blood pressure (mean, mmHg)	135.6 (95% CI 127–144)	137.2 (95% CI 133–141)
Diastolic blood pressure (mean, mmHg)	79.1 (95% CI 73.6–84.6)	81.2 (95%Ci 79–83.4)
D-dimer (median, ng/ml)	4400 (95% CI 4228–4400)	1169 (95% CI 1062–1376)
CRP (mean, mg/ml)	141.7 (95% CI 103–180)	84 (95% CI70.4–97.6)
White cell count (mean, ×10^9^/l)	9.95 (95% CI 8.5–11.3)	7.83 (95% CI 7.3–8.3)
Lymphocyte count (mean, ×10^9^/l)	1.23 (95% CI 0.88–1.58)	1.24 (95% CI 0.83–1.65)
Creatinine (mean, μmol/l)	86.5 (95% CI 75.7–97.3)	76.2 (95% CI 72.9–79.5)
Platelets count (mean, ×10^9^/l)	325 (95% CI 325–327)	323 (95% CI 322–324)
INR (mean)	1.23 (95% CI −0.48–2.94)	1.20 (95% CI 0.425–1.98)
Prothrombin time (mean, s)	12.8 (95% CI 11.1–14.5)	12.6 (95% CI 11.8–13.4)
Fibrinogen (mean, g/l)	6.26 (95% CI 4.55–7.97)	6.62 (95% CI 5.84–7.4)
ITU admissions (%)	7 (21%)	40 (25%)

INR: International normalized ratio; ITU: Intensive treatment unit; PE: Pulmonary embolism.

### Radiological parameters

Of the 33 patients who had a PE on CTPA imaging, 18 patients (55%) had bilateral PE’s and 15 (45%) had unilateral. About 15 patients (45%) had a segmental PE, 15 (45%) had a central PE and three (10%) had a sub-segmental PE. In total, 164 patients (85%) had radiological evidence of pneumonitis, representing 134 patients from the PE-negative group and 30 patients from the PE-positive group.

### Physiological parameters

The mean oxygen saturation at presentation, as measured by pulse oximetry, was lower in the PE-positive group in comparison to the PE-negative group (82.6% [95% CI: 81.5–83.7] vs 89.1% [95% CI: 87.4–90.8]; p < 0.001). The mean respiratory rate at presentation was higher in the PE-positive group in comparison to the PE-negative group (25.6 [95% CI: 23.9–27.3] vs 21.9 [95% CI: 21.2–22.7]; p < 0.001). There was no significant difference between heart rate, temperature and systolic and diastolic blood pressures at presentation between the PE-positive and -negative groups.

### Biochemical parameters

The median D-dimer was significantly higher in the PE-positive group (4400 ng/ml, [95% CI: 4228–4400]) in comparison with the PE-negative group (1169 ng/ml, [95% CI: 1062–1376]; t = 40.221; p < 0.01). The mean CRP and total WCC were also significantly greater in the PE-positive group (141 mg/l and 9.9 × 10^9^/l, respectively) in comparison with the PE-negative group (84.1 mg/l and 7.8 × 10^9^/l, respectively; p < 0.001). D-dimer and CRP concentration showed weak positive correlation (Spearman’s Rho: 0.304; p < 0.001). There was no significant difference between lymphocyte counts, creatinine levels, fibrinogen, platelets, INR and prothrombin time between the PE-positive and -negative groups.

**Figure 2.**
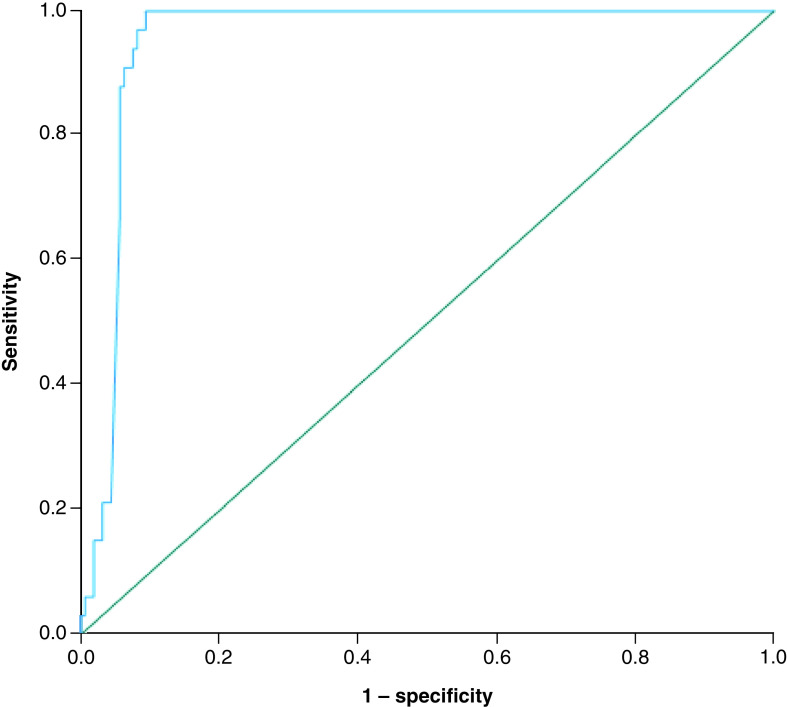
Receiver operating characteristic curve to determine the optimal cut-off value for D-dimer to predict the occurrence of pulmonary embolism on computerized tomographic pulmonary angiography in patients with COVID-19.

ROC curve analysis using Statistical Package for the Social Sciences software was performed to determine the optimal cut-off value for the D-dimer concentration to predict a CTPA positive for PE with high accuracy and precision. The ROC curve is seen in Figure 2. Area under the curve: 0.952 (95% CI: 0.922–0.982; p < 0.001). Using the Youden Index to determine the optimal cutoff, a D-dimer value of 2495 ng/ml was selected; Youden value: 0.906. This was verified using an Index of Union Calculation (Appendix 1). Using our dataset, a D-dimer cutoff of 2495 ng/ml gives a sensitivity of 100% (95% CI: 100–100), specificity of 90.62% (95% CI: 90.48–90.77), positive predictive value of 68.75% (95% CI: 68.33–69.17) and negative predictive value of 100% (95% CI: 100–100) for PE diagnosis. Patients with D-dimers >2495 ng/ml represented 24.87% of our sample.

### D-dimer, clot burden & clot location

Of the 33 PE-positive patients, there was no significant difference in D-dimer concentration between those with unilateral and those with bilateral PEs (U = 154; p = 0.509). There was also no significant difference in D-dimer concentration between those with subsegmental, segmental and central PEs (t = 3.186; p = 0.203). The calculated Wells score did not differ significantly between the positive and negative groups (1.28 [95% CI: 0.94–1.62] and 1.86 [95% CI: 1.59–2.13], respectively; p = 0.09). There was no relationship between laterality nor location or PE and Wells scores.

## Discussion

There is an unmet need for guidance on identifying patients that warrant further investigation for PE in COVID-19 pneumonitis. In our retrospective two center analysis, we show that D-Dimer concentration may be used to predict the presence of co-existent PE in patients with COVID-19 infection. In COVID-19 patients who underwent a CTPA, a D-dimer value above 2495 ng/ml was an excellent predictor of the presence of PE, with 100% sensitivity and 90.6% specificity. Values below 2495 ng/ml had 100% negative predictive value.

### Correlations with previous work

Our findings are in concordance with several studies which have proposed a D-Dimer threshold as an indicator of co-existent PE in COVID-19 and those that have demonstrated a correlation between D-Dimer values and mortality. This cut-off value slightly differed between studies. In a similar patient cohort, with a similar PE prevalence (27.2%), Mouhat *et al.* [13] found that a D-dimer concentration greater than 2590 ng/ml conferred a 17-fold increase in the risk of PE in 162 hospitalized patients with COVID-19 pneumonitis with a resultant sensitivity of 83.3% and specificity of 83.8%. Ventura-Diaz *et al.* [15] placed this threshold at 2903 ng/ml (resultant sensitivity 81%) in their retrospective cohort of 242 hospitalized patients with COVID-19 and a PE prevalence of 30%.

### Additional predictors of PE

We observed a significantly higher respiratory rate and lower oxygen saturations on admission in patients who were PE positive compared with the negative group. Kefale *et al.* [16] demonstrated a pooled prevalence of 33% of thrombo-embolism in hospitalized patients with COVID-19 and this was associated with elevated concentration of D-dimer. The risk of thrombo-embolism was found to be more prevalent in those who were admitted to the ICU, in keeping with our finding that PE was more prevalent in patients who were more hypoxic and had a high respiratory rate. Roncon *et al.* [17] presented similar findings, with a higher proportion of patients admitted to ICU with COVID-19 having a PE on CTPA compared with ward patients.

CRP and total WCC levels were also higher in our patients with PE. CRP levels have been shown to be predictive of more severe COVID-19 disease, thus fitting with work suggesting thromboembolic complications are more frequent in patients with more severe COVID-19 [18]. The increased thromboembolic risk in COVID-19 patients could be due to a procoagulant state generated by the severity of the infection [19] and the magnitude of the inflammatory response and that is probably the reason that D-Dimer levels are quite high in COVID-19 patients.

Our study showed a positive correlation between CRP and D-dimer concentration, comparable to previous studies [20,21]. Unlike existing work, we did not observe a relationship between increasing D-dimer levels and mortality [22–24]. This may reflect improving therapeutic options and increased clinical experience in the second wave of COVID-19 [25,26]. Furthermore, our study had median age of 67 yrs in patients with confirmed PE, which can also be contributory to a low death rate as previous studies clearly showed that age group more than 70 yrs of age has the highest risk of dying from COVID-19 [27].

Notably, our study finds that the Wells score, a widely utilized risk stratification tool for PE, correlated poorly with the presence of PE and may not be applicable in patients with COVID-19 pneumonitis. Recent studies concur with this finding, with a Wells score of >4 only being found in 33% of COVID-19 patients with subsequent PE [28].

### Strengths & limitations

Our proposed D-dimer cutoff is in line with three of the four previously published studies, validating use of such a figure and provides the best sensitivity and specificity characteristics of all the previously proposed cutoffs. Furthermore, ours is the only study to include data from two hospitals and to be conducted in latter ‘waves’ of the pandemic, where clinical acumen, diagnostics and treatments have progressed significantly since March 2020, when the other four studies were conducted. Given this, we feel that our work contributes significantly to the body of evidence existing in this area and advances previously published work with better test characteristics, a wider population and provides a representation of current management of COVID-19. Furthermore, the prevalence of PE detected in our study was in line with the Royal College of Radiologists data of 15.4–37.4% [29] and previous studies, suggesting generalizability of our results. Our reported D-dimer cutoff of 2495 ng/ml is in line with other threshold studies, supporting its use in clinical practice.

However, there are several limitations to our study. It is a retrospective analysis of patients admitted with COVID-19 who underwent a CTPA; therefore, there may have been selection bias, in other words, the patients selected for CTPA were suspected of having high pretest probability of PE. The sample size was small. We did not collect data on Doppler ultrasound of legs and; therefore, we cannot rule out deep vein thrombosis as the cause of elevated D-Dimers. Transthoracic echocardiography was not done in the present study to assess right ventricle (RV) function in COVID-19-associated PE patients.

Larger prospective studies are required to formulate a risk prediction tool for PE in COVID-19, which could incorporate a D-Dimer cutoff, respiratory rate, WCC, CRP and validated COVID-19 severity scores.

## Conclusion

We propose that even in the absence of other clinical signs of PE (such as unexplained syncope, ECG findings of right heart strain, hypoxia out of proportion to CXR findings), a D-dimer threshold of 2495 ng/ml could be used with high sensitivity and specificity to predict PE and hence the need for CTPA imaging in patients with COVID-19. In cases of clinical uncertainty, a high CRP, profound hypoxia and elevated respiratory rate should prompt consideration that the patient may have PE. We found that D-Dimer values do not correlate with clot burden and, therefore, cannot be considered for utilization in patients with hemodynamic instability who may be considered for thrombolysis. Further work is required to corroborate our findings and enable their dissemination into widespread clinical practice.

**Summary points**

- COVID-19 is a known risk factor for pulmonary embolism (PE). There is considerable overlap between symptoms of PE and COVID-19, complicating clinical PE detection. Furthermore, D-dimer levels are typically elevated in COVID-19, making their use challenging in determining which patients require computerized tomographic pulmonary angiography (CTPA) imaging.
- This retrospective multicenter study aims to determine an optimal D-dimer threshold for predicting PE in COVID-19 patients which maximizes sensitivity and specificity and, hence, can be used with confidence in clinical practice.
- A total of 193 patients met the inclusion criteria (COVID-19 positive, D-dimer levels measured at admission, CTPA scan performed). Of these, 33 cases of PE were detected (17%).
- Using receiver operating characteristic analysis (AUC = 0.952), we found an optimal D-dimer cutoff of 2495 ng/ml to predict/exclude PE. This level yields a sensitivity of 100% (95% CI: 100–100), specificity of 90.62% (95% CI: 90.48–90.77), positive predictive value of 68.75% (95% CI: 68.33–69.17) and negative predictive value of 100% (95% CI: 100–100) for PE diagnosis and is in line with previous similar work.
- We propose that in the absence of other clinical signs of PE, a D-dimer threshold of 2495 ng/ml could be used with high sensitivity and specificity to predict PE and hence the need for CTPA imaging in patients with COVID-19.
